# Social Determinants of Health as Predictors of Accelerated Alzheimer's Disease Biomarker Accumulation and Cognitive Deterioration

**DOI:** 10.1002/alz70860_104782

**Published:** 2025-12-23

**Authors:** Han Kyu Na, Han‐Kyeol Kim, Elena Tsoy, Katherine L. Possin, Victor Valcour, Gil D. Rabinovici, Chul Hyoung Lyoo, Hanna Cho

**Affiliations:** ^1^ Gangnam Severance Hospital, Yonsei University College of Medicine, Seoul, Korea, Republic of (South); ^2^ Wonju Severance Christian Hospital, Yonsei University Wonju College of Medicine, Wonju, Gangwon‐do, Korea, Republic of (South); ^3^ Memory and Aging Center, University of California San Francisco, San Francisco, CA, USA; ^4^ Global Brain Health Institute, University of California, San Francisco, San Francisco, CA, USA; ^5^ Global Brain Health Institute (GBHI), University of California San Francisco (UCSF); & Trinity College Dublin, San Francisco, CA, USA

## Abstract

**Background:**

Social Determinants of Health (SDOH), including education and socioeconomic status, are known to influence cognitive health and dementia risk in older adults. However, the mechanisms by which SDOH affect brain health, particularly Alzheimer's disease (AD) biomarkers, are not well understood. This study aimed to explore the impact of socioeconomic disparities on the progression of AD biomarkers over time.

**Methods:**

This retrospective study examined participants undergoing longitudinal evaluation of AD biomarkers: amyloid, tau, and neurodegeneration (ATN), using dual positron emission tomography (PET) with ^18^F‐Florbetaben and ^18^F‐Flortaucipir, brain MRI, and neuropsychological assessments. Participants were classified into low, middle, and high SDOH groups based on a composite score reflecting education, income, residential area, and comorbid vascular risk factors. Linear mixed models were used to assess changes in amyloid and tau accumulation and cognitive performance across SDOH groups, adjusting for age, sex, education, APOE genotype, and cognitive status.

**Results:**

The study included 185 participants (healthy controls, *n* = 77; mild cognitive impairment, *n* = 75; dementia, *n* = 33), with 73 participants (39.5%) testing positive for amyloid on PET scans. Cross‐sectional analyses showed no significant differences in amyloid or tau burden across the SDOH groups. Among amyloid‐positive individuals, the low SDOH group had a significantly faster rate of tau accumulation compared to the middle (*p* = 0.006) and high SDOH groups (*p* = 0.002). Additionally, the low SDOH group exhibited a more rapid decline in cognitive performance, particularly in language and visuospatial domains. Among amyloid‐negative participants at baseline, the low SDOH group showed faster amyloid accumulation (*p* = 0.0186) and greater decline in language function (*p* = 0.009) compared to the high SDOH group.

**Conclusions:**

These findings suggest that disadvantaged SDOH are associated with accelerated AD progression, including faster tau accumulation in amyloid‐positive individuals and more rapid amyloid accumulation in amyloid‐negative individuals. The results highlight the importance of incorporating SDOH in clinical trials and the need for targeted interventions for those in the low SDOH group.

